# Type 2 Diabetic Patients Adherence Towards Their Medications

**DOI:** 10.7759/cureus.6932

**Published:** 2020-02-10

**Authors:** Khalid A Alshehri, Talal M Altuwaylie, Ali Alqhtani, Albaraa A Albawab, Abdulrahman H Almalki

**Affiliations:** 1 Medicine, King Saud Bin Abdulaziz University for Health Sciences, Jeddah, SAU; 2 Family Medicine, Ministry of National Guard Health Affairs, Jeddah, SAU

**Keywords:** diabetes, medications, adherence, education, awareness, diabetes education, jeddah, saudi arabia, t2dm, dm

## Abstract

Given that type 2 diabetes mellitus (T2DM) has become a common noncommunicable disease that can begin in early life and has a significant effect on the quality of life, it is important to address treatment adherence. While various factors contribute to the development of T2DM, the primary cause is linked to unhealthy eating and lack of physical activity. Adhering to healthy eating and physical exercise is not generally well tolerated by patients with diabetes, and pharmacological treatments are often necessary. However, few studies exist that assess T2DM treatment adherence in Saudi Arabia. Therefore, we conducted a cross-sectional analytic study at Al Iskan, Al Waha, and Bahrah Primary Health Care Centers, National Guard Health Affairs, Jeddah, Saudi Arabia, to assess the adherence among patients with T2DM via a self-completed questionnaire after participant verbal and written consent. A total of 387 patients with T2DM were surveyed: 269 were males (69.5%) and 118 were female patients (30.5%). According to our findings, 265 (68.5%) participants reported adherence toward their medications, and 122 (31.5%) were not adherent. The most common reason for nonadherence was forgetting to take their medication. Nonadherence is associated with poor outcomes and lower quality of life. Therefore, additional studies and awareness campaigns are needed to identify and address the various causes of nonadherence to prevent further complications and decrease the overall burden of the disease.

## Introduction

Diabetes mellitus (DM) is a chronic, noncommunicable disease that is gradually increasing in incidence around the world. The total number of adults living with DM has increased nearly four times, rising from 108 million cases to 422 million cases as of 2014. More than half of these patients are uninformed about their condition, and many receive no remedy [1]. Moreover, in 2012, deaths attributed to DM totaled 1.5 million, and another 2.2 million deaths were associated with high blood glucose levels. Deaths due to DM are expected to rise, and DM may become the seventh leading cause of death in 2030 [2].

DM is characterized by a condition where the body cannot utilize glucose properly due to a defect in insulin production. Insulin is an essential hormone that metabolizes glucose, and a lack of insulin leads to hyperglycemia. DM is classified into two major types: type 1 DM (T1DM) and type 2 DM (T2DM). T1DM is characterized by an absence of insulin production caused by an autoimmune disease. T2DM results from insufficient production of insulin or from the body becoming resistant to its own produced insulin [3]. Furthermore, T2DM is the most common type of diabetes globally [4]. The third type of diabetes is gestational diabetes, which occurs only during pregnancy. A pregnant woman can recover from gestational diabetes after delivery but she will be more susceptible afterward to developing T2DM [3].

Lack of physical activity, poor dietary intake, and smoking are marked as major risk factors that increase the possibility of developing T2DM [5]. The treatment for T2DM includes pharmacological agents and lifestyle modification. Poor adherence to T2DM treatment leads to severe medical conditions such as gangrene, nephropathy, retinopathy, neuropathy, heart attack, and stroke [6].

We conducted this study to assess treatment adherence in patients with T2DM in Saudi Arabia, Jeddah, at National Guard for Health Affairs (NGHA). This study also sought to determine obstacles that lead to poor adherence and the factors that may improve patient compliance with treatment.

## Materials and methods

This cross-sectional study was conducted at Al Iskan, Al Waha, and Bahrah Primary Health Care Centers, NGHA, Jeddah, Saudi Arabia, from December 2017 to November 2018. The study included primary care patients diagnosed with T2DM. Patients were excluded if they had T1DM or did not have a diabetes diagnosis or did not consent to participate.

The study data were collected using a questionnaire (Table 1) consisting of three parts. The first part collected demographic characteristics, the second part collected T2DM diagnosis information, and the final part consisted of 20 yes or no questions to assess adherence to diabetes medications. Patient names were not collected to maintain confidentiality. This questionnaire was directly handed to the patients after obtaining verbal consent. The questionnaire was not shared with office staff or physician. 

**Table 1:** Questionnaire

Demographic data		
Age (years)		
Gender (male/female)		
Nationality		
Marital status		
Education		
Occupation		
Profile of diabetes mellitus		
Duration of diabetes mellitus		
Age at onset		
Family history of diabetes (yes/no)		
Adherence assessment		
Do you take the antidiabetes drugs as advised by your doctor? (yes/no)		
No, please tick the options [✓]		
Items	Yes	No
Lack of finance		
Feeling drug is not effective		
Interferes with my meal plan		
Taking them since many years		
I forget		
Side effects		
Feeling the dose given is high		
Complexity of drug regimen		
Multiple medications		
Poor family support		
Questions regarding diabetes practices		
Do you regularly monitor your blood glucose?		
Do you make your own modification in the dose of drugs prescribed?		
Do you make your own modification in the timing of antidiabetic drugs?		
Do you have good knowledge about antidiabetic medications prescribed to you?		
Do you know the importance of antidiabetic medication?		
Did your physician give information on diabetes?		
Did your physician give information on antidiabetic medications?		
Were you involved in treatment decisions?		
Do you feel comfortable to ask questions to your doctor?		

Data were analyzed using IBM SPSS Statistics for Windows, Version 23.0 (Armonk, NY: IBM Corp.). Participants were randomly selected in the study area at the time of data collection, and the sample size was calculated using Rao Online Software (Raosoft, Inc. Seattle, WA). The margin of error was 5%, with a 95% confidence level. This study was approved by our institutional review board.

## Results

The study included 387 patients with T2DM (269 males, 69.5%; 118 females, 30.5%). The mean age was 54±11 years. Most patients (n=348; 89.9%) were educated, and 39 patients (10.1%) were not educated. A total of 201 were employed (51.93%), 134 were retired (34.62%), and 52 (13.43%) were not employed. In addition, most participants (n=259; 66.9%) had a positive family history of DM. 

Most patients (n=265; 68.5%) reported adherence to their medications, and 122 (31.5%) reported that they did not adhere to their T2DM treatment. A variety of reasons were provided for nonadherence (Table 2), but the most common reason reported was forgetting to take their medication (n=82; 67.21%). Another major reason for nonadherence to T2DM treatment was fatigue with taking medications for a long time (n=61, 50%). A total of 54 participants (44.26%) reported that taking many medications prevented them from adherence toward their medications and 49 participants (40.16%) reported that the complexity of their treatment regimen was the reason for their nonadherence. Lack of family support (n=47; 38.52%), adverse side effects from medication (n=43; 35.24%), interference with their meal planning (n=43, 30.32%), feeling that their dose was too high (n=37; 30.32%), and feeling that the treatment was not effective (n=30; 24.59%) were also cited as reasons for nonadherence. A few participants (n=16; 4.13%) responded that lack of finances caused a lack of adherence.

**Table 2:** Study participant reasons for nonadherence

Reason	Answered yes, n (%)
Forget to take medication	82 (67.21%)
Use them for a long period of time	61 (50%)
Taking many medication	54 (44.26%)
Complexity of their treatment regimen	49 (40.16%)
Lack of family support	47 (38.52%)
Medication side effects	43 (35.24%)
treatment interferes with their meal plan	43 (35.24%)
Feel the dose is too high	37 (30.32%)
Feel the treatment is not effective	30 (24.59%)
Lack of finance	16 (13.11%)

More than half of the respondents (n=241; 62.3%) reported that they do not measure blood glucose levels regularly, and a majority (n=224; 57.9%) reported that they do not modify their daily doses of oral antihyperglycemic agents (OHA). A total of 209 patients (54%) reported that they adjust the timing of their doses. In addition, 227 patients (71.6%) have good knowledge of their condition. Also, most participants (n=304; 78.6%) know the importance of their medications, but 83 participants (21.4%) do not.

Most respondents (n=282; 72.9%) stated that their physicians give them good information about their condition. Also, most respondents (n=272; 70.3%) reported being satisfied with their physicians' information regarding their treatment, whereas 115 respondents (29.7%) reported dissatisfaction.

We found that 209 respondents (54%) indicated they were involved in treatment decisions, while 178 respondents (46%) were not. Most respondents (n=275; 71.1%) reported feeling comfortable to ask their doctors regarding their condition, whereas 112 respondents (28.9%) were not comfortable in asking about their condition (Table 3).

**Table 3:** Study participant diabetes practices

Question	Answered yes
Measure blood glucose regularly?	146 (37.7%)
Modify the dose of your medications?	162 (41.9%)
Modify the timing of your medications?	209 (54%)
Have good knowledge about the medications?	227 (71.6%)
Know the importance of your medications?	304 (78.6%)
Your physician gives you information about diabetes?	282 (72.9%)
Your physician gives you information about antidiabetic medications?	272 (70.3%)
Involved in treatment plan and decision?	209 (54%)
Feel comfortable to ask questions to your doctor?	275 (71.1%)

## Discussion

Adherence levels and the factors that influence adherence vary from study to study, and the variation could be due to differences in traditions, customs, and environmental factors among various societies and regions.

A systematic review conducted by Cramer in the United States assessed patients with diabetes’ adherence with oral hypoglycemic agents and insulin and reported that 36% to 93% of the patients taking OHA remained on their treatment for 6-24 months. For insulin adherence, Cramer estimated that adherence was 62% and 64% for patients on long-term and short-term insulin, respectively. She also reported that some of the patients omitted OHA doses and insulin injections before a clinical visit. She emphasized the need for further investigations about specific factors that might lead to an improvement in medication adherence. Our results are within the range of adherence reported by Cramer for OHA [7].

A similar study was carried out in Egypt by Heissam et al. in 2015 for OHA adherence in 372 participants [8]. Patients achieving a result of more than 75% on the Morisky-Green-Levine scale were considered as having good adherence, as score less than 50% was considered poor adherence, and a score between 50% and 75% was considered decent adherence. The study reported that among the participants, 26.1% were found to have good adherence, 47.9% had a decent adherence, and 26% had poor adherence, concluding that the rate of adherence was not acceptable, and patient education is a key factor for better health care management [8]. We found that 68.5% of the participants followed their medical plan as prescribed by their doctors, and 31.5% of them do not adhere to their medical plan, which is a higher level of adherence than that reported by Heissam.

Another study in three hospitals in central Ethiopia reported generally poor adherence and poor glycemic control in 41.8% of the participants [9]. Another study by Ibrahim et al. reported that 57% of the patients with diabetes take their medications as prescribed [10].

We found that, of nonadherent patients, the most common reason cited was patients forgetting to take their medications (67.2% of the nonadherent patients). Ibrahim et al. reported that the complications of the medications was the most common reason for nonadherence (63.3%) [10]. Wabe et al. reported a lack of finances (37.1%) and forgetting the medication (50.2%) were common reasons cited for nonadherence [9].

In our study, 44.26% of the participants reported that taking many medications interferes with adherence to their antidiabetic agents, which is a smaller percentage than that reported by Cramer (36%) for the same reason [7]. Cramer and Heissam reported that monotherapy had better adherence than multitherapy treatment plans [7,8].

Drug side effects were cited by 35.24% of the participants as reasons for poor adherence, which is similar to the proportion of respondents citing the same reason in the Wabe study (29.2%), but smaller than that reported by Heissam (65.43%) [8,9]. We found that some participants did not measure their blood glucose levels regularly, which was also reported by Heissam et al. and Harris [8,11].

In our study, more than half of the participants do not modify their dose. However, Ibrahim et al. reported that more than half (57%) of their sample indicated they take their medication as prescribed by their physicians [10]. Wabe et al. reported that a small proportion of their patients (20.8%) take their medications as prescribed by their health care providers [9].

Most of our respondents (71.6%) reported adequate knowledge about their condition, which was a larger percentage than that reported by Heissam et al., who reported that nearly half of their participants have adequate knowledge about the disease [8].

Our study had several limitations. The questionnaire is self-administered, which may have introduced participant bias. In order to avoid this bias, we measured glycosylated hemoglobin to assess the patients' condition. Another limitation was the refusal of some patients to participate in this study. 

## Conclusions

T2DM and its complications are becoming more common worldwide. Patients' adherence to their medications can minimize morbidity and mortality. The overall percentage of medication adherence among patients with T2DM is less than optimal for several reasons. The primary reason for suboptimal adherence was patient forgetfulness. Health awareness campaigns may play a vital role in improving patient adherence levels. Further studies are warranted to explore the impact of awareness campaigns on patient forgetfulness as a means to improve adherence to T2DM treatment.

## References

[1] Chan M (2017). Obesity and diabetes: the slow-motion disaster. Milbank Q.

[2] Emerging Risk Factors Collaboration, Sarwar N, Gao P (2010). Diabetes mellitus, fasting blood glucose concentration, and risk of vascular disease: a collaborative meta-analysis of 102 prospective studies. Lancet.

[3] American Diabetes Association (2020). Classification and diagnosis of diabetes: standards of medical care in diabetes-2020. Diabetes Care.

[4] Kaiser AB, Zhang N, Van der Pluijm W (2018). Global prevalence of type 2 diabetes over the next ten years (2018-2028). Diabetes.

[5] Bi Y, Wang T, Xu M (2012). Advanced research on risk factors of type 2 diabetes. Diabetes Metab Res Rev.

[6] Forbes JM, Cooper ME (2013). Mechanisms of diabetic complications. Physiol Rev.

[7] Cramer JA (2004). A systematic review of adherence with medications for diabetes. Diabetes Care.

[8] Heissam K, Abuamer Z, El-Dahshan N (2015). Patterns and obstacles to oral antidiabetic medications adherence among type 2 diabetics in Ismailia, Egypt: a cross section study. Pan Afr Med J.

[9] Wabe NT, Angamo MT, Hussein S (2011). Medication adherence in diabetes mellitus and self management practices among type-2 diabetics in Ethiopia. N Am J Med Sci.

[10] Ibrahim NK, Attia SG, Sallam SA, Fetohy EM, El-Sewi F (2010). Physicians' therapeutic practice and compliance of diabetic patients attending rural primary health care units in Alexandria. J Family Community Med.

[11] Harris MI, Cowie CC, Howie LJ (1993). Self-monitoring of blood glucose by adults with diabetes in the United States population. Diabetes Care.

